# Engineering decentralized electrodisinfection to sustain consistent chlorine generation under varying drinking water chloride content

**DOI:** 10.1016/j.apcato.2024.207012

**Published:** 2024-10

**Authors:** Aksana Atrashkevich, Sergi Garcia-Segura

**Affiliations:** Nanosystems Engineering Research Center for Nanotechnology-Enabled Water Treatment, School of Sustainable Engineering and the Built Environment, Arizona State University, Tempe, AZ 85287-3005, USA

**Keywords:** Electrochlorination, Active chlorine species, Chlorate, Perchlorate, Dimensional stable anode (DSA), Decentralized water treatment, Disinfection

## Abstract

*In situ* electrochlorination can offer an efficient and feasible solution to enable decentralized water disinfection. Unfortunately, there has been only a limited number of studies exploring single-pass flow cell systems with representative flowrates used at household level, particularly under varying chloride concentrations. This work aims to assess anode materials in a single pass and examine the impact of cross velocity, current density, and chloride concentration on various responses such as chlorine production and energy consumption. The primary objective is to determine whether the flow cell can achieve desirable chlorine levels under consistent operation while chloride content of water varies. Chlorine (Cl_2_/HOCl/OCl^−^), chlorine dioxide (ClO_2_) production, and toxic oxyanions (ClO_3_^−^, ClO_4_^−^) were assessed in a single pass setup utilizing different representative anodes including Ti/RuO_2_, Ti/IrO_2_, and Boron-doped diamond. Among these materials, the Ti/RuO_2_ anode emerged as the most suitable for effective chlorine generation while minimizing the formation of ClO_3_^−^ and ClO_4_^−^. The performance of *in situ* electrochlorination using the Ti/RuO_2_ anode in the flow cell revealed that cross velocity exerted the most significant influence on chlorine generation, while chloride content and current density primarily impacted energy consumption. Optimization of the operating parameters illustrated that stable chlorine concentrations ranging from 2 to 4 mg L^−1^ could be maintained even with significant fluctuations in chloride concentration from 50 to 250 mg L^−1^, resulting in a daily energy consumption of less than 0.07 kWh per treated volume of 634 L (i.e., < 0.11 Wh L^−1^). These experimental findings hold promise for advancing electrodisinfection systems to higher technological readiness level.

## Highlights

• Desired chlorine concentration for primary and secondary water disinfection achieved.
• Ti/RuO_2_ electrode most efficient for chlorine-based species generation.
• Boron-doped diamond exceeded limits of chlorate and perchlorate.
• Cross velocity is the most influential parameters on chlorine production.
• Energy consumption per day is much less than energy produced by one solar panel.

## Graphical abstract

**Figure:**
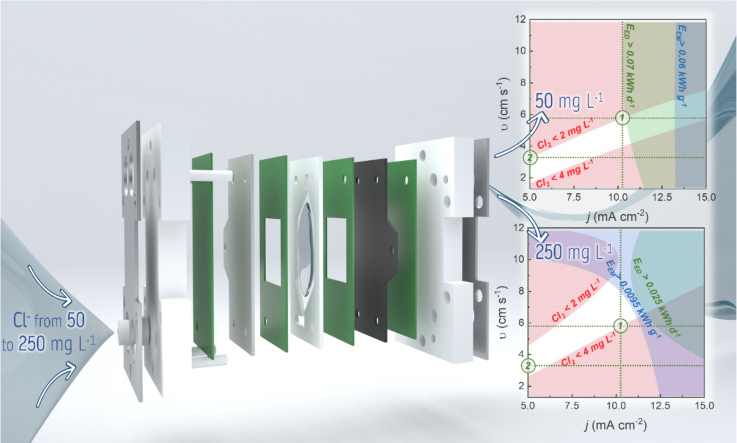

## 1 Introduction

Chlorination of water has reduced mortality due to water-borne diseases to nearly 0 % [1,2]. Access to clean water for all by 2030 has been identified by the United Nations (UN) as one of the sustainable development goals. However, 26 % of global population still do not have access to safely managed drinking water source [3]. Every year over 830,000 deaths are still associated to water-borne diseases [4]. Despite the great efforts, the UN informs that nearly 130 countries may not attain the holistic goal of ensuring sustainably managed drinking water resources unless strategies for faster implementation of water management solutions are provided. One of the major existing barriers towards this goal is the high capital cost required for centralized treatments and water distribution grids. To address this, emerging water management strategies should aim to provide easy access off-grid through modular, adaptative, and decentralized water systems. Electrified technologies meet these criteria and can become a feasible solution to provide safe water to final users at point-of-entry and point-of-use levels [[5], [6], [7], [8]]. Thus, electrified systems should be able to operate with low energy inputs. To enhance the accessibility of electrified water treatment, the maximum energy requirements of such systems should be below the minimum daily electrical outputs of a single solar panel, estimated to be approximately 1.0 kWh day^−1^ [9,10].

Electrogeneration of active chlorine species can be exploited as a competitive decentralized disinfection treatment due to ubiquity of chloride anions in water [11,12]. Electrochlorination can provide sustained and controlled dosing of active chlorine species, while avoiding the handling of hazardous chemicals. Oxidation of chloride ions into chlorine occurs through the transfer of two electrons at the anode surface according to the following reaction (1) [12,13]. Chlorine has low solubility in water and rapidly undergoes disproportionation yielding hypochlorous acid (HOCl) based on reaction (2). The speciation distribution of weak acid HOCl depends on the acid-base equilibria of reaction (3) with a p*K*_*a*_ of 7.5 at 25 °C.

(1) $$2{\mathrm{Cl}}^{-}\to {\mathrm{Cl}}_{2}+2e^{-}\;\;\;\;\;\;\;\;\;\;\;E^{o}=1.358\;V\;\mathrm{vs}\;\mathrm{SHE}$$

(2) $${\mathrm{Cl}}_{2}+H_{2}O\to \text{HOCl}+H^{+}+{\mathrm{Cl}}^{-}$$

(3) $$\text{HOCl}⇌H^{+}+{\mathrm{OCl}}^{-}\;\;\;\;\;\;\;\;\;\;\;{\mathrm{pK}}_{a}=7.5\;\mathrm{at}\;25^{{}^{\circ}}C$$

Active chlorine species enact primary disinfection and provide residual chlorine in water to sustain safe water for extended period of time. A recent study has demonstrated that electrochlorination offers superior microorganism inactivation efficiency compared to conventional chlorination. This enhanced performance is attributed to the high local chlorine concentration and low pH near the anode surface [14]. Analogous to centralized water treatment, decentralized *in situ* electrochlorination is the final disinfection step in the water treatment chain. Additional pretreatment strategies must be implemented to remove organics and minimize the formation of disinfection by-products. The modularity and small physical footprint of household electrochlorination systems allow for their integration with other decentralized water treatment processes (i.e., filtration, capacitive deionization, etc.). These multistage treatment chains can remove disinfection by-products precursors, achieving effective water disinfection with reduced formation of disinfection by-products, enabling water reuse strategies by regeneration of greywaters for secondary applications such as toilet flushing or irrigation.

Electrochlorination is extensively used by the chloro-alkali industry but has rarely been explored for scaled-down decentralized water disinfection. Electrocatalyst selection is a key aspect to ensure efficient electrogeneration of disinfecting species and maintaining sufficient chlorine residual. Most studies have explored electrochlorination technologies in batch reactors with promising results for organic pollutants abatement, and pathogens inactivation. However, the major barrier for translation is precisely the lack of studies exploring engineered systems that provide continuous flow treatment at realistic flow rates in single pass while using various chloride compositions found in diverse types of waters. Herein, the primary objective of this study is to determine if the flow cell can achieve required chlorine concentration for disinfection under consistent operating conditions while varying the inlet water chloride content. We evaluated the capabilities of an electrochemical flow cell to sustain continuous treatment of daily water consumption at household level up to 1296 L day^−1^, with various dilute chloride concentrations. Additionally, we examined the criteria for electrocatalyst selection, which was focused not only on the accumulation of active chlorine species but also considered the risks of disinfection by-product formation. Analysis of engineering parameters allowed us to evaluate energy requirements and guide optimization strategies for the efficient operation of electrochemically-driven systems within the energy range provided by a single solar panel (i.e., < 1.0 kWh day^−1^). The optimization of the electrochemical flow cell enabled the identification of two operational modes characterized by high and low water consumption. The operational parameters of these modes ensure the sustainable production of chlorine concentration with low energy consumption, even when the chloride concentration fluctuates by a factor of five, ranging from 50 to 250 mg L^−1^.

## 2 Materials and methods

### 2.1 Chemicals and solutions

Sodium chloride (NaCl, purity >99 %) used as electrolyte was supplied by Sigma-Aldrich. Active chlorine was analyzed using *N*,*N*-diethyl-*p*-phenylene diamine (DPD) reagent supplied by HACH for colorimetric method. The DPD reagent was used to quantify ClO_2_, after suppressing active chlorine through addition of glycine. All solutions were prepared with ultrapure water provided from an ultrapure water system Elga Water with resistivity >18.2 MΩ cm at 20 °C.

### 2.2 Electrochemical experiments

**Fig. 1:**
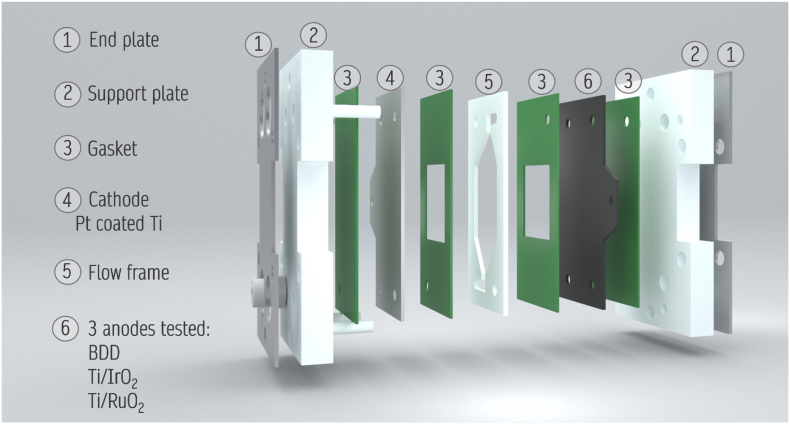
Exploded view at the Micro Flow Cell “ElectroCell” with surface area of electrodes of 10 cm^2^ and 0.4 cm electrode gap distance.

Fig. 1 illustrates the ElectroCell (Sweden) electrochemical flow cell employed during electrochlorination. Electrolysis was conducted under galvanostatic operating mode using a power supply TENMA 72–2710 by defining the applied current density ranging from 5 mA cm^−2^ up to 15 mA cm^−2^. The cell was operated under flow-by mode in a single pass of solution using a peristaltic pump DIGI-STALTIC Cole Parmer Instrument Co. set at different flows to attain cross velocities' values from 1.3 cm s^−1^ up to 11.9 cm s^−1^. In this work, cross velocity is defined as flow rate divided by surface area of the cell perpendicular to the flow direction 1.26 cm^2^ (i.e., 3.16 cm × 0.4 cm). The electrochemical cell was equipped with two parallel electrodes with a geometric area of 10 cm^2^ (i.e., 3.16 cm × 3.16 cm) each and located in parallel maintaining a 0.4 cm electrode gap distance. The cathode consisted of a platinum coated titanium plate, whereas different anode materials were evaluated as electrocatalysts. Boron-doped diamond (BDD) was used as representative “non-active” electrocatalytic anode material. Two dimensional stable anodes (DSA®) with differentiated electrocatalytic behavior were evaluated as representative “active” electrodes: Ti/RuO_2_ and Ti/IrO_2_. These electrode materials have been widely employed in the chloro-alkali industry and have demonstrated sustained production of reactive chlorine species during the electrode operational life of ≥10 years [15]. All electrodes were supplied by ElectroCell. The total volume within the electrochemical cell compartment was 4.0 mL and remained constant during all experiments. Samples were collected at the cell outlet after a minimum of one hundred cell volumes passed through the cell and potential was stable to ensure steady-state electrochemical transformation given the residence time in the cell.

### 2.3 Analytical instruments and performance evaluation

The water pH and conductivity were measured with a Thermo Scientific Orion Star A221 pH-meter and A322 conductivity meter, respectively. The outlet water pH was within the neutral range from 6.5 to 8.5, which is typical for drinking water [16]. The conductivity of tested water varied from 200 to 950 mS cm^−1^, corresponding to the amount of added NaCl. The concentrations of generated active chlorine (Cl_2_/HOCl/OCl^−^) and chlorine dioxide (ClO_2_) were quantified using DPD methods equivalent to colorimetric Standard Method 4500-Cl G [17] and 4500-ClO_2_ D [18], respectively. Absorbance of 530 nm wavelength was quantified through spectrophotometry using a HACH DR 6000 UV–vis. Ionic species chlorate (ClO_3_^−^) and perchlorate (ClO_4_^−^) were quantified by ion chromatography Dionex ICS-5000. Both oxyanions were evaluated utilizing a column Dionex AS−18 and KOH eluent gradient that started at 12 mM and ramped to 45 mM.

The formation efficiency of active chlorine species was evaluated in terms of Faradaic efficiency (FE) Eq. (4). The FE is an electrocatalytic figure-of-merit that describes the efficiency of charge transfer to enable the reaction of interests. The FE defines the percentage of number of electrons consumed for active chlorine generation relative to the total charge consumption [19].

(4) $$\mathrm{FE}\left(\%\right)=\frac{q_{\mathrm{reaction}}}{q_{\mathrm{total}}\;}\times 100\%=\frac{n\;F\;N_{i}\;}{\;I\;t}\times 100\%,$$

where $q_{\mathrm{reaction}}$ is the charge consumed in the reaction of interest (C); $q_{\mathrm{total}}$ is the total charge consumed during electrochemical process (C); *n* is the amount of electrons required per mole of product for a complete conversion; *F* is the Faraday constant (96,485C mol^−1^); $N_{i}$ is the amount of product generated during the electrolysis (mol); *I* is the applied current intensity (A); t is the hydraulic residence time (HRT) of water within the electrochemical flow cell.

The electric energy per mass of generated active chlorine (*E*_EM_) during operation was computed according to Eq. (5) [20].

(5) $$E_{\mathrm{EM}}\left(\mathrm{kWh}\;g^{-1}\right)=\frac{P\;}{\;Q\;\left(C_{o}-C_{t}\right)},$$

where *P* is the rated power (kW); *Q* is the flowrate (*L* *h*^−1^); *C*_*o*_ *and C*_*t*_ are the initial (influent) and final (effluent) concentrations of active chlorine (g L^−1^). The rated power is estimated from the product of applied current (A) and the cell potential (V).

The electrical energy consumption per day (*E*_ED_) at household level was calculated based upon Eq. (6). Despite electrochemical systems may be likely operated under on-off cycles based on intermittent greywater generation, it is assumed herein a sustained treatment over 24 h. Thus, *E*_ED_ requirements are overestimated under a conservative approach.

(6) $$E_{\mathrm{ED}}\left(\mathrm{kWh}\;{\mathrm{day}}^{-1}\right)=P\;\frac{24\;\mathrm{hours}\;}{\mathrm{day}}$$

The electrical energy consumption per volume (*E*_EV_) was calculated according to Eq. (7).

(7) $$E_{\mathrm{EV}}\left(\mathrm{kWh}\;L^{-1}\right)=\frac{P\;}{Q}\;$$

### 2.4 Response surface methodology

Response surface methodology was applied to establish the impact of operating parameters on *in situ* chlorine electrogeneration. The experiments were designed following the Box-Behnken method. Three influential factors were defined: chloride concentration in the range from 50 mg L^−1^ to 250 mg L^−1^, applied current density in the range from 5 mA cm^−2^ to 15 mA cm^−2^, and cross velocity from 1.3 cm s^−1^ to 11.9 cm s^−1^. According to the Box-Behnken design, the independent variables had three coded levels set as *x*_*i*_ = −1, 0, 1 with corresponding values summarized in Table 1. The relations between each response and independent variables were established based upon the second-order model described by Eq. (8):

(8) $$Y=\beta_{0}+\sum_{i=1}^{k}\beta_{i}x_{i}+\sum_{i=1}^{k}\beta_{\mathrm{ii}}x_{i}^{2}+\sum_{i=1}^{k}\sum_{i\#j=1}^{k}\beta_{\mathrm{ij}}x_{i}x_{j}+\varepsilon ,$$

where *Y* is the response measured; $\beta_{0}$ is a regression coefficient; $\beta_{i}$, $\beta_{\mathrm{ii}}$ and $\beta_{\mathrm{ij}}$ are regression coefficients for linear, quadratic and interaction effect, respectively. The index *k* indicates the number of the variables selected (i.e., 3), the *x*_*i*_ and *x*_*j*_ are different independent variables, and ε is the random error. Experiments at the central point (0,0,0) were conducted in triplicate and allowed to estimate the pure error. The runs were performed in a random manner to minimize the effect of unexplained variability on the observed responses due to systematic errors. The polynomial models were statistically validated using JMP and Minitab software.

**Table 1:** Coded levels and empirical values for the response surface methodology analysis.

Run	Coded levels			Parameters tested			Registered responses		
	x_1_	x_2_	x_3_	X_1_^a^	X_2_^b^	X_3_^c^	${\mathrm{Cl}}_{2}$ (mg L^−1^)	*E*_EM_ (kWh g^−1^)	*E*_ED_ (kWh day^−1^)
1	0	1	-1	6.6	15	50	2.7	0.065	0.1260
2	-1	1	0	1.3	15	150	13.75	0.023	0.0461
3	1	0	1	11.9	10	250	1.66	0.009	0.0204
4	-1	-1	0	1.3	5	150	4.66	0.011	0.0072
5	0	-1	-1	6.6	5	50	0.59	0.04	0.0170
6	0	0	0	6.6	10	150	2.62	0.014	0.0257
7	0	-1	1	6.6	5	250	1.15	0.007	0.0060
8	1	-1	0	11.9	5	150	0.51	0.012	0.0079
9	-1	0	1	1.3	10	250	11.2	0.01	0.0168
10	-1	0	-1	1.3	10	50	6.6	0.049	0.0470
11	0	0	0	6.6	10	150	2.06	0.024	0.036
12	0	1	1	6.6	15	250	4.72	0.011	0.0367
13	1	1	0	11.9	15	150	2.36	0.02	0.0619
14	0	0	0	6.6	10	150	2.52	0.014	0.0259
15	1	0	-1	11.9	10	50	0.98	0.057	0.0720

a Cross velocity, cm s^−1^.

b Current density, mA cm^−2^.

c Chloride concentration, mg L^−1^.

## 3 Results and discussion

### 3.1 Selection of anode material for *in situ* electrochlorination

The nature of the anode material stands as one of the most influential parameters in electrified water treatment technologies. Anodic material influences the lifetime of the systems, capital expenditures, and operational costs. However, the most significant impact lies in their electrocatalytic control over product and by-product formation [[21], [22], [23], [24]]. Previous studies on electrochlorination have predominantly utilized batch-type reactors with small volumes and long hydraulic residence times, resulting in high charge transfer per unit volume. Evaluation in batch reactor systems may lead to a substantial overestimation of chlorine production and the accumulation of undesired by-products (i.e., ClO_3_^−^, ClO_4_^−^) beyond the maximum contaminant levels for drinking water. In this context, assessing electrode materials while operating flow-by reactor under single-pass mode with realistic hydraulic retention times is of utmost importance to evaluate system competitiveness for *in situ* disinfection. Anodic materials are typically categorized based on the oxidation states available on their surface. “Active” anodes exhibit higher oxidation states, where adsorbed hydroxyl radicals (•OH) might chemically interact with the electrode surface (i.e., chemisorption). In contrast, at “non-active” anodes, the formation of higher oxides does not occur, resulting in weaker physical interactions between •OH and the electrode surface (i.e., physisorption). Consequently, •OH radicals are *quasi-* released into the solution and tend to present a higher oxidation capacity that those chemisorbed [25]. Herein, we explore the use of Ti/RuO_2_ and Ti/IrO_2_ as two representative “active” anodes, and BDD as the main representative of “non-active” anodes. Electrochlorination was conducted in a single pass electrochemical flow-by reactor with an HRT of 2.4 s at applied current density of 15 mA cm^−2^ with an inlet concentration of chloride of 150 mg L^−1^, representing an average chloride concentration commonly found in environmental waters and greywaters [11,26,27].

**Fig. 2:**
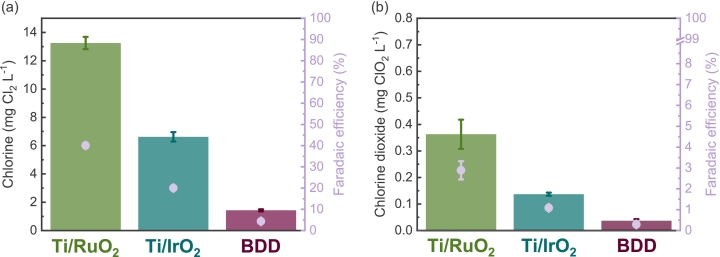
(a) Active chlorine and (b) chlorine dioxide generation (columns) and corresponding Faradaic efficiencies (circles) after single pass of electrochemical flow cell equipped with different anode materials (Ti/RuO_2_; Ti/IrO_2_, and BDD) and Ti as a cathode at chloride concentration of 150 mg L^−1^ and current density of 15 mA cm^−2^ at cross velocity of 1.3 cm s^−1^ (flow rate of 0.1 L min^−1^; HRT of 2.4 s).

The electrocatalytic performance was benchmarked in terms of active chlorine species (Cl_2_/HOCl/OCl^−^) and chlorine dioxide (ClO_2_). As depicted in Fig. 2 (a), even within a single pass with 2.4 s of HRT, the Ti/RuO_2_ anode achieved the highest active chlorine evolution value of 13.3 mg L^−1^, which was twice as high as that obtained using a Ti/IrO_2_ electrode (i.e., 6.6 mg L^−1^) and nearly ten times larger than with BDD (i.e., 1.4 mg L^−1^). Our results align with a previous study that reported higher chlorine evolution when using pure RuO_2_ as compared to pure IrO_2_ anode when both electrocatalytic coatings were manufactured through an identical thermal decomposition process. This increased chlorine evolution was attributed to several factors, including a lower oxygen/metal atomic ratio, lower binding energies of the *d*-derived *t*_2g_ valence bands in RuO_2_, and the lower resistance of RuO_2_, compared to IrO_2_ [28]. Using Ti/RuO_2_ anode, water pH increased from 6.0 to 8.4, while pH values of 8.3 and 7.3 were obtained using Ti/IrO_2_ and BDD, respectively. The distribution of chlorine species is defined by the p*K*_*a*_ of 7.5 of the species HOCl/OCl^−^ and therefore a function of pH. At a pH of 8.4 species distribution results in HOCl/OCl^−^ ratio of 0.11/0.89 for Ti/RuO_2_, pH 8.3 results in 0.14/0.86 ratio for Ti/IrO_2_, and pH 7.3 in 0.39/0.61 distribution for BDD. Higher pH resulted from suppressed oxygen evolution reaction, as chlorine generation competes with the oxygen evolution reaction. As Fig. 2 (a) illustrates, Ti/RuO_2_ anode achieved the highest Faradaic efficiency for chlorine generation of 40 %, flowed by Ti/IrO_2_ at 20 % and BDD at 4.3 %. A similar trend among anodes material was observed for ClO_2_ generation. The formation of ClO_2_ might occur through chemical and electrochemical reactions, following Eqs. (9), (10), (11), (12), (13), (14), (15) as described in literature [23,29,30].

(9) $${\mathrm{Cl}}^{-}+2H_{2}O\to {\mathrm{ClO}}_{2}+4H^{+}+5e^{-}\;\;\;\;\;\;\;\;\;\;\;E^{o}=1.599\;V\;\mathrm{vs}\;\mathrm{SHE}$$

(10) $${\mathrm{Cl}}^{-}+2H_{2}O\to {\text{HClO}}_{2}+3H^{+}+4e^{-}\;\;\;\;\;\;\;\;\;\;\;E^{o}=1.570\;V\;\mathrm{vs}\;\mathrm{SHE}$$

(11) $$\frac{1}{2}{\mathrm{Cl}}_{2}+2H_{2}O\to {\text{HClO}}_{2}+3H^{+}+3e^{-}\;\;\;\;\;\;\;\;\;\;\;E^{o}=1.628\;V\;\mathrm{vs}\;\mathrm{SHE}$$

(12) $$\text{HOCl}+H_{2}O\to {\text{HClO}}_{2}+2H^{+}+2e^{-}\;\;\;\;\;\;\;\;\;\;\;E^{o}=1.645\;V\;\mathrm{vs}\;\mathrm{SHE}$$

(13) $${\text{HClO}}_{2}⇌{\mathrm{ClO}}_{2}^{-}+H^{+}\;\;\;\;\;\;\;\;\;\;\;pK_{a}=1.94$$

(14) $${\mathrm{ClO}}_{2}^{-}\to {\mathrm{ClO}}_{2}+e^{-}\;\;\;\;\;\;\;\;\;\;\;E^{o}=0.954\;V\;\mathrm{vs}\;\mathrm{SHE}$$

(15) $${\text{HClO}}_{2}\to {\mathrm{ClO}}_{2}+H^{+}+e^{-}\;\;\;\;\;\;\;\;\;\;\;E^{o}=1.277\;V\;\mathrm{vs}\;\mathrm{SHE}$$

For BDD electrodes, the formation of Cl_2_ and ClO_2_ may also occur involving generation of radical species as intermediates following Eqs. (16), (17), (18) [31,32].

(16) $${\mathrm{Cl}}^{-}\to {\mathrm{Cl}}^{•}+e^{-}\;\;\;\;\;\;\;\;\;\;\;E^{o}=2.432\;V\;\mathrm{vs}\;\mathrm{SHE}$$

(17) $${\mathrm{Cl}}^{•}+{\mathrm{Cl}}^{•}\to {\mathrm{Cl}}_{2}$$

(18) $${\mathrm{Cl}}^{•}+O_{2}\to {\mathrm{ClO}}_{2}$$

Fig. 2 (b) shows that among tested electrodes, the Ti/RuO_2_ reached the highest ClO_2_ production and corresponding Faradaic efficiency of 2.9 %, 1.1 % and 0.3 % were obtained using Ti/IrO_2_ and BDD, respectively. This aligns with another study that demonstrated a higher ClO_2_ production using Ti/RuO_2_ compared to Ti/IrO_2_ [33]. The generation of chlorine dioxide within maximum residual disinfection level (MRDL) is advantageous for *in situ* disinfection as it possesses a higher oxidation capacity over a broad pH range [34]. Furthermore, chlorine dioxide produces fewer disinfection organic by-products such as trihalomethanes (THMs) and haloacetic acids (HAAs) than free chlorine [[35], [36], [37]]. Nonetheless, the MRDL established by the Environmental Protection Agency (EPA) is lower for chlorine dioxide (i.e., 0.8 mg L^−1^) than for active chlorine (i.e., 4 mg L^−1^). This lower value is set due to the relatively rapid formation of unacceptable levels of inorganic by-products such as chlorite (ClO_2_^−^) and chlorate anions (ClO_3_^−^) when ClO_2_ is present at high concentrations and/or under alkaline conditions [[38], [39], [40], [41]]. With Ti/RuO_2_ as an anode, even at large chlorine production (i.e., 13.3 mg L^−1^), the chlorine dioxide generation (i.e., 0.36 mg L^−1^) was still significantly below the MRDL. As the results demonstrate, Ti/RuO_2_ exhibits superiority among the anode materials in terms of favorable chlorine-based species evolution in a single pass of flow cell.

Apart from the formation of desirable species, the generation of toxic oxyanions such as chlorate (ClO_3_^−^) and perchlorate (ClO_4_^−^) occurs during electrochlorination. The World Health Organization (WHO) has established guideline values for chlorate at 700 μg L^−1^ and for perchlorate at 70 μg L^−1^ in drinking water. Consumption of ClO_3_^−^ and ClO_4_^−^ above these recommended levels is suspected to inhibit thyroid function [42,43]. Electrochemical formation of ClO_3_^−^ might occur through several sequential pathways of chloride and chlorine oxidation according Eqs. (19), (20) [29].

(19) $${\mathrm{ClO}}_{2}+H_{2}O\to {\mathrm{ClO}}_{3}^{-}+2H^{+}+e^{-}\;\;\;\;\;\;\;\;\;\;\;E^{o}=1.152\;V\;\mathrm{vs}\;\mathrm{SHE}$$

(20) $${\text{HClO}}_{2}+H_{2}O\to {\mathrm{ClO}}_{3}^{-}+3H^{+}+2e^{-}\;\;\;\;\;\;\;\;\;\;\;E^{o}=1.214\;V\;\mathrm{vs}\;\mathrm{SHE}$$

Using BDD electrode, formation of ClO_3_^−^, besides sequential direct oxidation of Cl^−^, might involve the contribution from radical species Eqs. (21), (22), (23), (24) [44,45].

(21) OH•+OCl−→OCl•+OH−

(22) OH•+OCl•→ClO2−+H+

(23) OH•+ClO2−→ClO2•+OH−

(24) OH•+ClO2•→ClO3−+H+

**Fig. 3:**
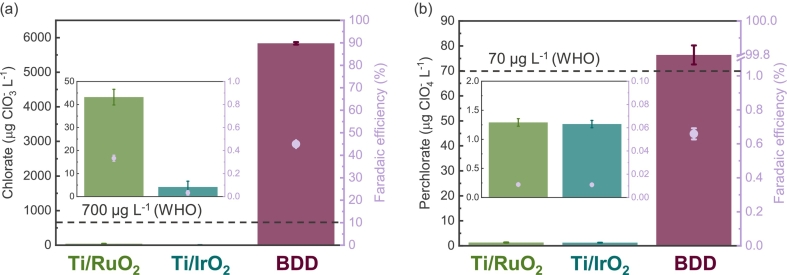
(a) Chlorate and (b) perchlorate generation (columns) and corresponding Faradaic efficiencies (circles) in a single pass of electrochemical flow cell equipped with different anode materials (Ti/RuO_2_; Ti/IrO_2_, and BDD) and Ti as a cathode under chloride concentration of 150 mg L^−1^ and current density of 15 mA cm^−2^ at cross velocity of 1.3 cm s^−1^ (flow rate of 0.1 L min^−1^; HRT of 2.4 s).

As shown in Fig. 3 (a), the generation of chlorate using both DSA electrodes were negligible compared to the guideline concentration for drinking water of 700 μg L^−1^. Chlorate production using Ti/RuO_2_ reached 43 μg L^−1^, while 4 μg L^−1^ was detected when utilizing the Ti/IrO_2_ anode. These generated concentrations correspond to negligible Faradaic efficiencies. In contrast, chlorate concentration using BDD as an anode exceeded the recommended concentration for drinking water by more than eight times in a single pass (i.e., 5835 μg L^−1^), with Faradaic efficiency of chlorate formation reaching 45 %.

The formation of ClO_4_^−^ using DSA electrodes may occur through the direct sequential oxidation of ClO_3_^−^ Eq. (25). For BDD anode, the oxidation of ClO_3_^−^ to ClO_4_^−^ might proceed through multiple pathways including Eqs. (25), (26), (27), (28) [29,32,[46], [47], [48]]:

(25) $${\mathrm{ClO}}_{3}^{-}+H_{2}O\to {\mathrm{ClO}}_{4}^{-}+2H^{+}+2e^{-}\;\;\;\;\;\;\;\;\;\;\;E^{o}=1.189\;V\;\mathrm{vs}\;\mathrm{SHE}$$

(26) ClO3−+OH•→ClO4−+H++e−

(27) $${\mathrm{ClO}}_{3}^{-}\to {\mathrm{ClO}}_{3}^{•}+e^{-}\;\;\;\;\;\;\;\;\;\;\;E^{o}=2.38\;V\;\mathrm{vs}\;\mathrm{SHE}$$

(28) ClO3•+OH•→ClO4−+H+

It has been reported that the BDD anode predominantly generates ClO_4_^−^ as a final product in batch type reactors [46,49]. Fig. 3 shows that in a single pass using BDD as an anode primary ClO_3_^−^ was produced with a value of 5835 μg L^−1^, while only 73.4 μg L^−1^ of ClO_4_^−^ was detected. These results indicate that the step of oxidation of ClO_3_^−^ into ClO_4_^−^ was not completed in a single pass under used settings. However, even this diminutive amount of 73.4 μg L^−1^ exceeded the recommended limit of perchlorate (i.e., 70 μg L^−1^). Conversely, both DSA electrodes generated negligible ClO_4_^−^ content of 1.3 μg L^−1^, which corresponded to negligible Faradaic efficiencies for ClO_4_^−^ formation. Faradaic efficiency for ClO_4_^−^ using BDD was near 0.7 %. The chloride concentration in water plays a determining role in the formation of toxic oxyanions while using BDD as an anode. Reduction in inorganic by-products generation may be achieved through precise control of operational variables based on inlet water characteristics [48,50,51]. Nevetherless, Ti/RuO_2_ exhibited the highest selectivity for chlorine generation compared to Ti/IrO_2_, while negligible Faradaic efficiencies were observed for the formation of toxic inorganic by-products compared BDD. Based on these data, it was concluded to investigate the effect of operating parameters of electrochemical flow cell and chloride content on *in situ* electrochlorination using Ti/RuO_2_ as an anode.

### 3.2 Impact of operating variables on *in situ* electrochlorination

To explore the influence of operating parameters, their interactions, and quadratic effects on *in situ* electrochlorination, specifically focusing on chlorine production and energy consumption, we utilized the Box-Behnken design. The Box-Behnken design, being a spherical design, facilitates the evaluation of system through minimizing the number of experimental runs while assessing variable interaction and synergies [52]. In this study, we assessed the most influential operating parameters affecting chlorine production and energy consumption, namely, chloride concentration, cross velocity, and applied current.

Considering dilute chloride concentrations that are typically present in freshwaters is crucial while scaling up electrochlorination for disinfection. Earlier studies on *in situ* electrochlorination have primarily focused on waters with extremely high salt content measured in grams per liter. The use of extreme concentrations that are not representative of real inlet water qualities may hinder our understanding and assessment of electrochlorination competitiveness for disinfection. Ideally, the concentration of chloride of inlet water should not surpass the maximum concentration level (MCL) of chloride for drinking water (i.e., 250 mg L^−1^) [53,54], otherwise that water would be unfit for consumption by the decentralized treatment final users. Furthermore, both the anionic water composition and chloride concentration are fluctuating parameters, even within the same water source, which might impact on electrochlorination performance. Thus, the variability of dilute chloride concentrations in waters must be taken into account while assessing the electrochemical treatment efficiency. We selected chloride concentrations in waters near real potable water conditions, ensuring it did not exceed the MCL for drinking water (i.e., 50–250 mg L^−1^). The ranges of cross velocity were also selected to mimic domestic run for electrochlorination system (i.e., 1.3–11.9 cm s^−1^ or 144–1296 L day^−1^) considering the ranged values of water consumption per household [55,56]. Current densities in the range of 5 to 15 mA cm^−2^ were chosen to maintain feasible energy consumption for an average user. Table 1 shows the design variables and observed empirical responses during the experiments.

The preliminary effect summary (*S1*) for three model responses, which includes all terms of second-order models, revealed that only interaction term of cross velocity and chloride concentration (i.e., x_1_x_2_) and the quadratic term of current density (i.e., x_2_x_2_) did not have a statistically significant impact on the responses. Consequently, it was decided not to incorporate these insignificant terms into the models (*S2*). The resulting regression equations for generated chlorine, energy consumption per mass, and energy consumption per day are presented below in Eqs. (29), (30), (31), respectively:

(29) $${\mathrm{Cl}}_{2}\;\left(\mathrm{mg}\;L^{-1}\right)=2.24–1.38{\text{8x}}_{1}+0.75{\text{7x}}_{2}+0.0074x_{3}+0.1020x_{1}x_{1}–0.000016x_{3}x_{3}–0.0683x_{1}x_{2}–0.001849x_{1}x_{3}+0.0007{\text{3x}}_{2}x_{3}$$

(30) $$E_{\mathrm{EM}}\;\left(\mathrm{kWh}\;g^{-1}\right)=0.04992+0.0004{\text{9x}}_{1}+0.003079x_{2}–0.000537x_{3}–0.000001x_{1}x_{1}+0.000001x_{3}x_{3}–0.0000{\text{4x}}_{1}x_{2}–0.000011x_{2}x_{3}$$

(31) $$E_{\mathrm{ED}}\;\left(\mathrm{kWh}\;{\mathrm{day}}^{-1}\right)=-0.0254+0.0011{\text{6x}}_{1}+0.0107{\text{4x}}_{2}–0.000209x_{3}-0.000116x_{1}x_{1}+0.000001x_{3}x_{3}+0.000143x_{1}x_{2}–0.000039x_{2}x_{3}$$

**Fig. 4:**
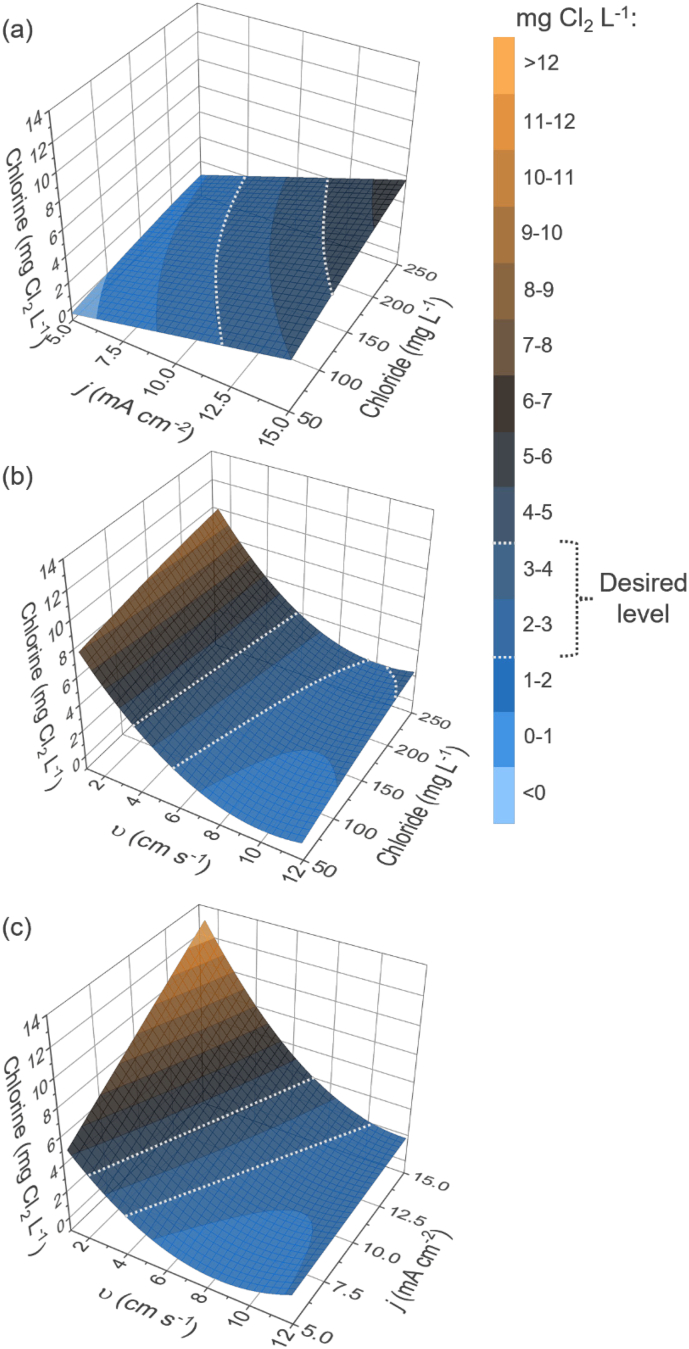
Response surfaces of generated active chlorine as functions of (a) chloride concentration and applied current density in the electrochemical flow cell at cross velocity of 6.6 cm s^−1^ (flow rate of 0.5 L min^−1^ or 720 L day^−1^; HRT of 0.5 s); (b) chloride concentration and cross velocity at current density of 10 mA cm^−2^; (c) cross velocity and applied current density at chloride concentration 150 mg L^−1^. The area in the chlorine response highlighted by the dotted white line indicates the target chlorine level of 2 to 4 mg L^−1^.

Fig. 4 (a) shows that altering chloride concentration by a factor of five, ranging from 50 to 250 mg L^−1^, induced a mild linear increase in active chlorine generation. A similar linear correlation between chloride content variation and chlorine production was observed at diverse cross velocities, as illustrated in Fig. 4 (b). The linear trend in chlorine response to chloride concentration fluctuation suggests minimal interaction effect of other variables with chloride concentration. As expected, higher levels of chloride concentration correspond to increased chlorine evolution. However, it is noteworthy that the five-fold increase in dilute chloride content did not exert a drastic influence on chlorine generation.

Elevated current densities led as well to higher concentration of generated chlorine. Fig. 4 (b) depicts that shift in current density from 5 to 15 mA cm^−2^ across various chloride concentrations (Fig. 4 (b)) and different cross velocities (Fig. 4 (c)) resulted in linear increase in chlorine, indicating mild interaction effects. In contrast, altering cross velocities from 1.3 cm s^−1^ to 11.9 cm s^−1^ led to a substantial change in chlorine. The impact of cross velocity on chlorine generation might be simply attributed to changes in charge transfer. Higher cross velocities led to reduced charge transfer and substantial decrease in chlorine formation. Curvatures were observed with varying cross velocities at various chloride concentrations (Fig. 4 (b)) and current densities (Fig. 4 (c)), showing the impact of interaction. Indeed, the effect tests (*S3*) confirm that, besides main effects, the interaction effect of cross velocity and current density as well as the quadratic effect of cross velocity influence the chlorine response model significantly.

For *in situ* electrochlorination systems at household level achieving a chlorine production range of 2 to 4 mg L^−1^ might be desirable to insure primary and secondary water disinfection. The lower threshold of the range aligns with the WHO recommendation for household water disinfection, aiming for approximately 2 mg L^−1^ at Nephelometric turbidity units (NTU) < 10 [57]. The upper threshold of 4 mg L^−1^ corresponds to MRDL according to EPA [58]. The data suggests that *in situ* electrochlorination might be a suitable solution for decentralized disinfection of waters with different dilute chloride concentrations. Five-fold difference in chloride content did not drastically change the active chlorine production. Pareto plot of estimates (*S6*) demonstrates that among the studied parameters and their ranges, chloride concentration causes the least influential main effect on chlorine production, while cross velocity holds the most substantial influence.

It is important to highlight that the tested water composition did not contain other co-existing anions besides chloride. It has been reported that co-existing anion species may inhibit chlorine generation by blocking active sites on the electrodes surfaces while using DSA materials as anodes [12,[59], [60], [61]]. However, one of the recent studies found no significant influence of co-existing anions such as SO_4_^2−^ and HCO_3_^−^ on electrochlorination while operating in a single pass and using graphite anode [62]. Further in-depth studies are needed to explore the potential effects of co-existing anions on electrochlorination. Additionally, hard water containing high concentrations of divalent cations, particularly Mg^2+^, can negatively impact electrolysis by causing scaling due to elevated pH at the cathode surface [7,63]. This is a limiting factor in practical applications and should be accounted for. To mitigate cathode scaling, strategies such as reverse polarization or water pretreatment that reduces divalent cations content in hard waters should be employed.

The reported energy consumption values during *in situ* electrochlorination vary in order of magnitude depending on the different reactor types and operating parameters considered in literature [[64], [65], [66]]. Most studies in literature have been conducted using batch reactors or batch recirculation. Evaluation of energy consumption in batch type reactors with large interelectrode gap, high residence time, and small treated volumes can result in overestimations of energy requirements, which does not provide a realistic picture of potential applicability and competitiveness of electrified disinfection technologies. Therefore, it makes it challenging to benchmark the feasibility of decentralized electrodisinfection under household settings against other concepts available in the market. In this study, electrical energy consumptions per mass and per day of operation, typically processed by decentralized system, were incorporated to estimate the feasibility of technology in a more profound way. It is crucial to emphasize that when evaluating electrified technologies for decentralized settings, the assessment of energy consumed per day is of utmost importance, as it directly determines the feasibility of the operation.

**Fig. 5:**
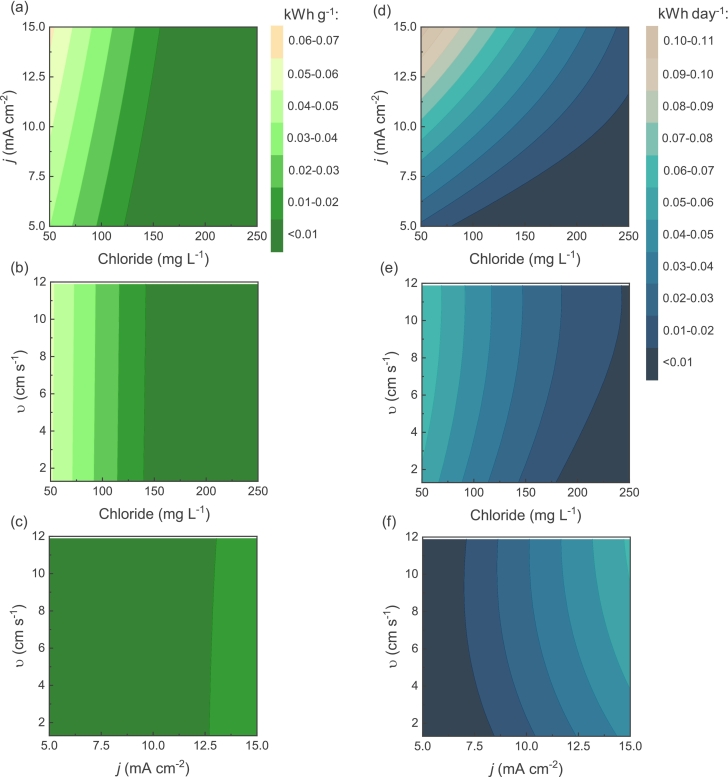
Contour plots of electrical energy consumption per mass (*E*_EM_) as functions of (a) chloride concentration and applied current density in the electrochemical flow cell at cross velocity of 6.6 cm s^−1^ (flow rate of 0.5 L min^−1^ or 720 L day^−1^; HRT of 0.5 s); (b) chloride concentration and cross velocity at current density of 10 mA cm^−2^; (c) cross velocity and applied current density at chloride concentration 150 mg L^−1^. Contour plots of electrical energy consumption per day (*E*_ED_) as functions of (d) chloride concentration and applied current density at cross velocity of 6.6 cm s^−1^; (e) chloride concentration and cross velocity at current density of 10 mA cm^−2^; (f) cross velocity and applied current density at chloride concentration 150 mg L^−1^.

Fig. 5 illustrates the counter plots of electrical energy consumption per gram of generated active chlorine and per day of operation. Overall, the energy consumption per mass and per day exhibit similar profiles. The most feasible operating conditions are caused by an increase in chloride concentration and a decrease in applied current. In contrast to chlorine response, variations in cross velocities have almost no impact on both energy consumptions. Energy consumption per unit mass depends on the operational power and inversely on both flow rate and chlorine generation Eq. (5). As increased flow rate significantly reduces chlorine generation, their product has little effect on the overall energy used. Energy consumption per day is directly proportional to the operational power Eq. (6). Effect tests for energy consumption per mass (*S4*) confirms that current density, chloride concentration, the interaction term of current density and chloride, and the quadratic term of chloride concentration significantly influence energy consumption per mass. Analogously, energy consumption per day is influenced by the same terms. As observed in Fig. 5 (a), (b), and (c), chloride concentration has the most influential impact on energy consumption per mass response. Elevated chloride concentration not only causes more efficient chlorine evolution, but also influences water conductivity and, consequently, cell potential. The Pareto plot of estimates for energy consumption per mass (*S7*) confirms that chloride content is the most significant parameter for energy consumption per mass, followed by its quadratic term, current density, and the interaction term of current density with chloride. It is important to note that the chloride concentrations used in this study (i.e., 50 to 250 mg L^−1^) correspond to relatively low water conductivities (i.e., 200 to 950 mS cm^−1^). Treating more complex water matrices containing various ions could result in decreased energy consumption due to elevated water conductivities. However, as discussed above, this might also induce additional challenges, such as the blockage of active sites of the anode by co-existing anions and reduce chlorine production. In the case of energy consumption per day of operation, the most significant parameter influencing the energy consumption per day is current density (Fig. 5 (d), (e), and (f)). This is supported by Pareto plot (*S8*), which highlights the significant impact of current density, followed by chloride concentration, interaction term of current density with chloride, and quadratic term of chloride. As expected, a higher degree of interaction is observed between current density and chloride concentration, as both variables affect the cell voltage. Since energy consumption per day is directly related to applied current and cell voltage, the applied current and chloride concentration play a pivotal role during cell operation.

The results on electric energy consumption per day at household level demonstrates that the highest energy consumption per day within the investigated ranges of operating parameters was below 0.13 kWh day^−1^, which is only one hundredths of the average daily consumption in the U.S [67,68]. Overall, the findings of influential nature of operating parameters on chlorine evolution and energy consumption responses yield valuable insights into cell operation. The lower impact of chloride concentration, compared with high influence of cross velocity on chlorine production, underscores the possibility of identifying optimal operating parameters even with variations in chloride concentration. Minimizing energy consumption can be achieved by reducing the applied current. Decentralized electrochlorination for enhanced disinfection may be plausible in a wide range of water matrix composition, including greywater reuse. The following section discusses the optimal operating parameters encompassing all response models.

### 3.3 Operating parameters optimization

Optimization of the electrochemical cell was conducted by focusing on three key responses including the concentration of generated chlorine, energy consumption per mass, and energy consumption per day. The objective was to attain a chlorine concentration range from 2 to 4 mg L^−1^ while minimizing both energy consumptions. The target range was selected based on recommended thresholds to enable primary and secondary water disinfection [57,58]. The optimization of such electrochemical systems might be a challenging task. Even if the system's operating parameters are initially set to be the optimum, maintaining consistent efficiency becomes problematic due to the fluctuating chemical composition of water under real-world conditions. Moreover, adaptive electrochemical cell operating conditions for various water compositions may require costly setups to continuously monitor water composition and adjust operational parameters accordingly. Such an approach might inflate the cost of the system and its maintenance. Hence, it becomes imperative to identify optimal operating conditions that remain stable across different water compositions.

**Fig. 6:**
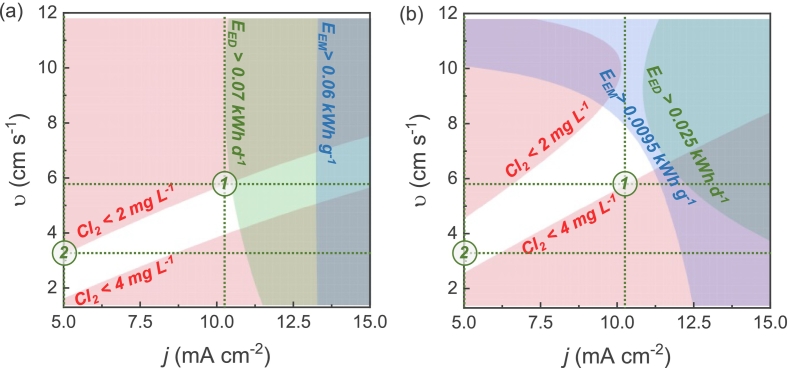
Overlaid contour plots of generated chlorine (red), electrical energy consumption per mass (blue), and electrical energy consumption per day (green) as function of current density and cross velocity at (a) chloride concentration of 50 mg L^−1^ and (b) chloride concentration of 250 mg L^−1^. Numbers 1 and 2 stand for 1 and 2 operation modes at high and low water consumption, respectively. (For interpretation of the references to colour in this figure legend, the reader is referred to the web version of this article.)

In this study, we identified two optimal operating modes that obviate the need for adjustment of cell conditions across a wide range of water chloride concentrations. Figs. 6 (a) and (b) depict overlaid counter profiles of chlorine, energy consumption per mass, and energy consumption per day for two chloride concentrations of 50 to 250 mg L^−1^, respectively. The red field represents the boundary conditions for the chlorine response, where undergeneration (i.e., Cl_2_ < 2 mg L^−1^) and overgeneration (i.e., Cl_2_ > 4 mg L^−1^) of active chlorine occur. The fields of energy consumption indicate that energy consumption in the selected area exceeds the mentioned level. Modes 1 and 2 stand for two operation modes designed for high and low water consumption scenarios, ensuring minimum energy consumption. As illustrated in Fig. 6 (a) and (b), when larger volumes of water require treatment, the system might operate at higher flow rates and correspondingly higher current densities to reach desired chlorine concentration. This initial operational mode (i.e., mode 1) was identified under conditions where it is possible to achieve the desired chlorine level (i.e., 2 to 4 mg L^−1^) for both chloride concentrations (i.e., 50 and 250 mg L^−1^) while treating the largest volume of water under used settings. Conversely, under conditions where lower water consumption is applicable (i.e., mode 2), operation might proceed at reduced cross velocities and current densities, thereby yielding cost savings through diminished energy consumption.

Table 2 highlights that running at higher cross velocity at mode 1, resulting in total water consumption per day of 634 L, requires operational current density of 10.2 mA cm^−2^ to achieve the desired chlorine production across varied chloride concentrations. In contrast, when lower water consumption is considered, a reduction in current density to 5 mA cm^−2^ may yield reduced energy consumptions, given that current density exerts the most pronounced influence on energy consumption per day. However, it is important to note that employing a storage tank with *in situ* electrochlorination might be necessary to achieve desired log of microorganism inactivation. This would increase the contact time of water with chlorine, thereby elevating the *Ct* value (i.e., the product of chlorine concentration and contact time).

**Table 2:** Characteristics of the operation modes.

Chloride concentration (mg L^−1^)	High water consumption Mode 1 (5.8 cm s^−1^ or 634 L d^−1^ at 10.2 mA cm^−2^)				Low water consumption Mode 2 (3.2 cm s^−1^ or 350 L d^−1^ at 5.0 mA cm^−2^)			
	**Chlorine** (mg L^−1^)	***E***_**EM**_ (kWh g^−1^)	***E***_**ED**_ (kWh d^−1^)	***E***_**EV**_ (kWh L^−1^)	**Chlorine** (mg L^−1^)	***E***_**EM**_ (kWh g^−1^)	***E***_**ED**_ (kWh d^−1^)	***E***_**EV**_ (kWh L^−1^)
50	2.0	0.0532	0.0681	0.0001074	2.1	0.0403	0.0159	0.0000454
250	4.0	0.0093	0.0210	0.0000331	3.3	0.0073	0.0096	0.0000274

The establishment of two distinct operational modes servicing to both high and low water demands, while minimizing energy consumption and ensuring the attainment of desired chlorine levels across varying water compositions, underscores the robustness of electrochlorination for water disinfection. Furthermore, the operational simplicity of these modes remains evident, facilitating straightforward operation even at fluctuating water compositions.

### 3.4 Models validation

The statistical significance of regression Eqs. (29), (30), (31) was confirmed using analysis of variance (ANOVA) and Fisher's distribution (*F*-test). The *F*-values of the models represent the ratio between the mean squares (MS) of the models and the MS of the errors. The *F*-values obtained for the models significantly exceeded the critical *F*-value at a 95 % confidence level, which was determined based on the models' degrees of freedom (i.e., *F*_0.05, 7,7_ = 3.79). Consequently, all *p*-values were below 0.05 (Table 3), rejecting the null hypothesis and indicating a significant contribution of the models. To evaluate the adequacy of the models, a lack-of-fit test was performed. The obtained *F*-values for lack-of-fit were lower than the critical *F*-value for lack-of-fit at a 95 % confidence level (i.e., *F*_0.05, 5,2_ = 19.30), resulting in *p*-values for lack-of-fit exceeding α = 0.05 for all regression equations. These findings suggest that the null hypothesis for lack-of-fit cannot be rejected, indicating no evidence that the regressions fail to fit the empirical data.

**Table 3:** Analysis of variance, lack of fit, and the summary fit.

		Chlorine (mg L^−1^)				*E*_EM_ (kWh g^−1^)				*E*_ED_ (kWh d^−1^)			
Source	DF	SS	MS	*F*-Value	*p*-Value	SS	MS	*F*-Value	*p*-Value	SS	MS	*F*-Value	*P*-Value
Model	7	204.71846	29.2455	24.3221	0.0002	0.00495	0.000707	45.4820	0.0001	0.01339	0.001912	22.5878	0.0003
Error	7	8.41698	1.2024			0.00011	0.000016			0.00059	0.000085		
Lack-of-Fit	5	8.23858	1.6477	18.4722	0.0521	0.00004	0.000008	0.2528	0.9067	0.00052	0.000105	3.017	0.2675
Pure Error	2	0.17840	0.0892			0.00007	0.000033			0.00007	0.000035		
Total	14	213.13544				0.00506				0.01398			
		R^2^ = 0.9605		R^2^_adj_ = 0.9210		R^2^ = 0.9785		R^2^_adj_ = 0.9570		R^2^ = 0.9576		R^2^_adj_ = 0.9152	

DF: Degree of freedom; SS: Sum of Squares; MS: Mean Square.

**Fig. 7:**
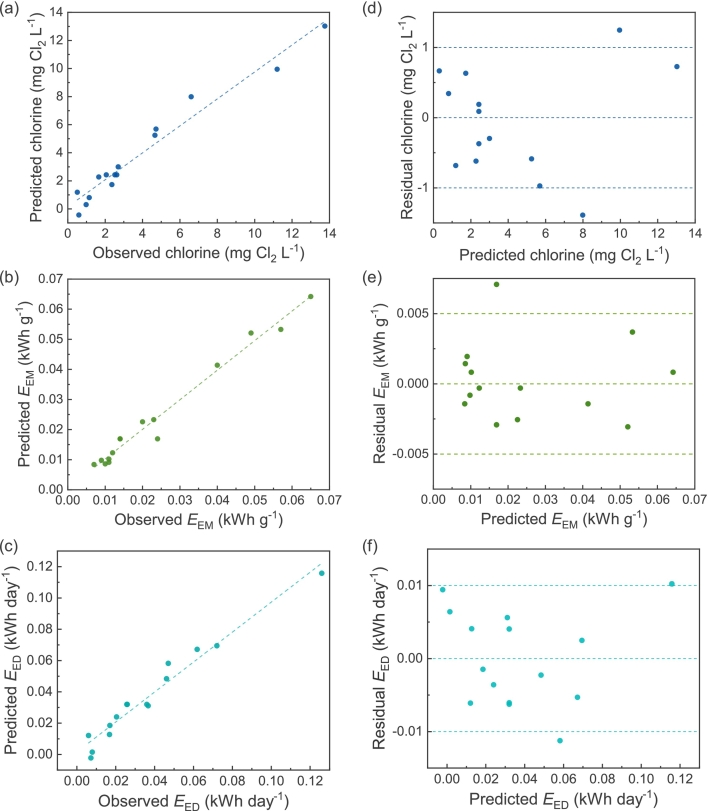
Observed versus predicted for (a) chlorine; (b) energy consumption per mass (*E*_EM_); and (c) energy consumption per day (*E*_ED_). Residual versus predicted for (d) chlorine; (e) energy consumption per mass; and (f) energy consumption per day.

To assess the alignment between predicted values and actual outcomes, we examined graphs comparing predicted versus observed values (*S9*). Figs. 7 (a), (b), and (c) demonstrate that the predicted and observed values in the graphs are fairly randomly scattered and closely aligned, suggesting that the predicted values match well with the observed ones. The random distribution of points indicates no notable pattern in model predictions. We further assessed model adequacy by examining residual versus predicted graphs. Figs. 7 (d), (e), and (f) show that the points in these graphs are also randomly scattered and relatively close to a mean residual value of zero. This random scattering and lack of systematic pattern suggest constant variance in the models and the absence of bias.

The R^2^ values, which measures proportion of total variability explained by the model, for chlorine response and both energy consumption models, are close to unity (Table 3) [52]. Moreover, the adjusted R^2^_adj_ values, which adjusted to the “size” of the models and decrease if nonsignificant terms are added to the model, are high for all the investigated responses and also close to unity [52]. These results confirm the adequacy of the obtained models. Consequently, these findings indicate that the models are statistically significant and capable of predicting responses.

## 4 Conclusion

In this work, the chloride-based species (i.e., Cl_2_/HOCl/OCl^−^, ClO_2_, ClO_3_^−^, and ClO_4_^−^) generation was evaluated using Ti/RuO_2_, Ti/IrO_2_, and BDD anodes in a single pass of flow cell. The result showed superiority of the Ti/RuO_2_ anode under selected ranges of operating parameters compared to Ti/IrO_2_, and BDD materials, particularly in terms of the active chlorine and chlorine dioxide production. Additionally, Ti/RuO_2_ formed negligible concentration of toxic oxyanions (i.e., ClO_3_^−^, ClO_4_^−^). Therefore, the performance of electrochemical flow cell for *in situ* electrochlorination was assessed using Ti/RuO_2_ anode, focusing on active chlorine production and energy consumption. Through response surface methodology, we evaluated the impact of operating parameters such as current density, chloride concentration, and cross velocity. The significance of the obtained models was statistically validated. The response model on active chlorine production using Ti/RuO_2_ anode revealed that cross velocity exerted the most significant influence among the investigated parameters, while chloride concentration had the least impact on chlorine generation. Even with a five-fold decrease in chloride concentration in the influent, active chlorine production did not change in a drastic way. In contrast to chlorine evolution response, chloride concentration and current density were the primary influencers of energy consumption. The maximum daily energy consumption was obtained at the lowest chloride content and the maximum current, however, being below 0.13 kWh, which is lower than the output of a single solar panel. Two operation modes were proposed for the cell operation for both high and low water consumption. At these two modes of operation, stable and desirable concentration of chlorine production might be achieved while operating with low energy input even despite drastic changes in chloride content. These results underscore the robustness of *in situ* electrochlorination for water disinfection even at various chemical compositions. Further in-depth research on various water matrices and disinfection mechanisms are needed to negotiate challenges during the treatment.

## CRediT authorship contribution statement

**Aksana Atrashkevich:** Writing – review & editing, Writing – original draft, Visualization, Methodology, Investigation, Formal analysis, Data curation, Conceptualization. **Sergi Garcia-Segura:** Writing – review & editing, Writing – original draft, Visualization, Validation, Supervision, Resources, Project administration, Methodology, Funding acquisition, Formal analysis, Data curation, Conceptualization.

## Declaration of competing interest

Sergi Garcia-Segura is an Editor of the journal but was not involved in the peer review or handling of this paper.

## Data Availability

Data will be made available on request.

## Appendix A Supplementary data

Supplementary material Image 1
