# Supplementary material for: Engineering decentralized electrodisinfection to sustain consistent chlorine generation under varying drinking water chloride content
Source: Appl Catal O Open. 2024 Oct;195:None. doi: 10.1016/j.apcato.2024.207012 (PMC11482661; doi:10.1016/j.apcato.2024.207012)
Supplement: Supplementary file 1 — Supplementary material [file mmc1.docx]

**Supplementary information**

**Engineering decentralized electrodisinfection to sustain consistent chlorine generation under varying drinking water chloride content.**

Aksana Atrashkevich, Sergi Garcia-Segura^a^*

^a^Nanosystems Engineering Research Center for Nanotechnology-Enabled Water Treatment,

School of Sustainable Engineering and the Built Environment, Arizona State University, Tempe, AZ 85287-3005, USA

*Article submitted to be published in*

*Applied Catalysis O: Open*

Corresponding author:

*e-mail: Sergio.garcia.segura@asu.edu (Dr. Sergi Garcia-Segura)

***S1. Effect summary for chlorine, energy consumption per day, and energy consumption per mass responses.***

| **Source** | **Log Worth** | ***p-*value** |
| --- | --- | --- |
| Chloride concentration | 4.588 | 0.00003 |
| Cross velocity | 4.034 | 0.00009 |
| Current density | 3.582 | 0.00026 |
| Chloride concentration $\times$ Chloride concentration | 2.888 | 0.00130 |
| Cross velocity $\times$ Cross velocity | 2.658 | 0.00220 |
| Current density $\times$ Chloride concentration | 2.135 | 0.00733 |
| Cross velocity $\times$ Current density | 1.893 | 0.01278 |
| Cross velocity $\times$ Chloride concentration | 1.020 | 0.09552 |
| Current density $\times$ Current density | 0.417 | 0.38283 |

In case when the multiple responses are present, JPM provides only one effect summary table. The table shows the minimum *p*-values among all *p*-values from multiple responses for that factor. Log Worth of models is -log_10_(*p*-value). This modification of *p*-value simplifies data analysis since Log Worth that is higher than 2 is statistically significant at the 0.01 level. As can be observed from Table *S1*, only 2 last terms are not statistically significant. Thus, it was concluded to remove the last two terms from the models. Removal of terms applied to all models.

***S2. Effect summary for chlorine, energy consumption per day, and energy consumption per mass responses after not significant terms were removed.***

| **Source** | **Log Worth** | ***p-*value** |
| --- | --- | --- |
| Chloride concentration | 5.969 | 0.00000 |
| Cross velocity | 4.640 | 0.00002 |
| Current density | 3.975 | 0.00004 |
| Chloride concentration $\times$ Chloride concentration | 3.639 | 0.00023 |
| Cross velocity $\times$ Cross velocity | 2.824 | 0.00150 |
| Current density $\times$ Chloride concentration | 2.206 | 0.00377 |
| Cross velocity $\times$ Current density | 1.883 | 0.01310 |

***S3. Effect tests for chlorine response.***

| **Source** | **DF** | **Sum of Squares** | ***F* Ratio** | **Prob > *F*** |
| --- | --- | --- | --- | --- |
| Cross velocity | 1 | 117.81125 | 97.9780 | <0.0001 |
| Current density | 1 | 34.52805 | 28.7153 | 0.0011 |
| Chloride concentration | 1 | 7.72245 | 6.4224 | 0.0390 |
| Cross velocity $\times$ Current density | 1 | 13.10440 | 10.8983 | 0.0131 |
| Current density $\times$ Chloride concentration | 1 | 0.53290 | 0.4432 | 0.5269 |
| Cross velocity $\times$ Cross velocity | 1 | 30.51225 | 25.3756 | 0.0015 |
| Chloride concentration $\times$Chloride concentration | 1 | 0.09971 | 0.0829 | 0.7817 |

***S4. Effect tests for energy consumption per mass.***

| **Source** | **DF** | **Sum of Squares** | ***F* Ratio** | **Prob > *F*** |
| --- | --- | --- | --- | --- |
| Cross velocity | 1 | 0.00000312 | 0.2010 | 0.6674 |
| Current density | 1 | 0.00030013 | 19.3081 | 0.0032 |
| Chloride concentration | 1 | 0.00378450 | 243.4708 | <0.0001 |
| Cross velocity $\times$ Current density | 1 | 0.00000400 | 0.2573 | 0.6275 |
| Current density $\times$ Chloride concentration | 1 | 0.00011025 | 7.0928 | 0.0323 |
| Cross velocity $\times$ Cross velocity | 1 | 4.94505e-8 | 0.0032 | 0.9566 |
| Chloride concentration $\times$Chloride concentration | 1 | 0.00074207 | 47.7399 | 0.0002 |

***S5. Effect tests for energy consumption per day.***

| **Source** | **DF** | **Sum of Squares** | ***F* Ratio** | **Prob > *F*** |
| --- | --- | --- | --- | --- |
| Cross velocity | 1 | 0.00025425 | 3.0029 | 0.1267 |
| Current density | 1 | 0.00676285 | 79.8752 | 0.0001 |
| Chloride concentration | 1 | 0.00414505 | 48.9568 | 0.0002 |
| Cross velocity $\times$ Current density | 1 | 0.00005700 | 0.6733 | 0.4390 |
| Current density $\times$ Chloride concentration | 1 | 0.00153272 | 18.1028 | 0.0038 |
| Cross velocity $\times$ Cross velocity | 1 | 0.00003909 | 0.4617 | 0.5187 |
| Chloride concentration $\times$Chloride concentration | 1 | 0.00057164 | 6.7516 | 0.0355 |

***S6. Pareto plot of estimates for chlorine response.***

| **Term** | ***t* Ratio** | |
| --- | --- | --- |
| Cross velocity | -9.90 |  |
| Current density | 5.36 |  |
| Cross velocity $\times$ Cross velocity | 5.04 |  |
| Cross velocity $\times$ Current density | -3.30 |  |
| Chloride concentration | 2.53 |  |
| Current density $\times$ Chloride concentration | 0.67 |  |
| Chloride concentration $\times$Chloride concentration | -0.29 |  |

***S7. Pareto plot of estimates for energy consumption per mass.***

| **Term** | ***t* Ratio** | | |
| --- | --- | --- | --- |
| Chloride concentration | | -15.60 |  |
| Chloride concentration $\times$Chloride concentration | | 6.91 |  |
| Current density | | 4.39 |  |
| Current density $\times$ Chloride concentration | | -2.66 |  |
| Cross velocity $\times$ Current density | | -0.51 |  |
| Cross velocity | | 0.45 |  |
| Cross velocity $\times$ Cross velocity | | -0.06 |  |

***S8. Pareto plot of estimates for energy consumption per day.***

| **Term** | ***t* Ratio** | | |
| --- | --- | --- | --- |
| Current density | | 8.94 |  |
| Chloride concentration | | -7.00 |  |
| Current density $\times$ Chloride concentration | | -4.25 |  |
| Chloride concentration $\times$Chloride concentration | | 2.60 |  |
| Cross velocity | | 1.73 |  |
| Cross velocity $\times$ Cross velocity | | 0.82 |  |
| Cross velocity $\times$ Current density | | -0.68 |  |

***S9. Observed and predicted values using the Box-Behnken design.***

| Run | Parameters tested | | | Observed | | | Predicted | | |
| --- | --- | --- | --- | --- | --- | --- | --- | --- | --- |
|  | **X_1_^a^** | **X_2_^b^** | **X_3_^c^** | $\mathbf{Cl}_{\mathbf{2}}$  **(mg L^-1^)** | ***E*_EM_**  **(kWh g^­-1^)** | ***E*_ED_**  **(kWh day^­-1^)** | $\mathbf{Cl}_{\mathbf{2}}$  **(mg L^-1^)** | ***E*_EM_**  **(kWh g^­-1^)** | ***E*_ED_**  **(kWh day^­-1^)** |
| 1 | 6.6 | 15 | 50 | 2.7 | 0.065 | 0.1260 | 3.00 | 0.0642 | 0.1158 |
| 2 | 1.3 | 15 | 150 | 13.75 | 0.023 | 0.0461 | 13.02 | 0.0233 | 0.0484 |
| 3 | 11.9 | 10 | 250 | 1.66 | 0.009 | 0.0204 | 2.28 | 0.0098 | 0.0240 |
| 4 | 1.3 | 5 | 150 | 4.66 | 0.011 | 0.0072 | 5.25 | 0.0091 | -0.0022 |
| 5 | 6.6 | 5 | 50 | 0.59 | 0.04 | 0.0170 | -0.43 | 0.0414 | 0.0185 |
| 6 | 6.6 | 10 | 150 | 2.62 | 0.014 | 0.0257 | 2.43 | 0.0169 | 0.0320 |
| 7 | 6.6 | 5 | 250 | 1.15 | 0.007 | 0.0060 | 0.81 | 0.0084 | 0.0121 |
| 8 | 11.9 | 5 | 150 | 0.51 | 0.012 | 0.0079 | 1.19 | 0.0123 | 0.0015 |
| 9 | 1.3 | 10 | 250 | 11.2 | 0.01 | 0.0168 | 9.95 | 0.0086 | 0.0127 |
| 10 | 1.3 | 10 | 50 | 6.6 | 0.049 | 0.0470 | 7.99 | 0.0521 | 0.0582 |
| 11 | 6.6 | 10 | 150 | 2.06 | 0.024 | 0.036 | 2.43 | 0.0169 | 0.0320 |
| 12 | 6.6 | 15 | 250 | 4.72 | 0.011 | 0.0367 | 5.69 | 0.0102 | 0.0311 |
| 13 | 11.9 | 15 | 150 | 2.36 | 0.02 | 0.0619 | 1.73 | 0.0226 | 0.0672 |
| 14 | 6.6 | 10 | 150 | 2.52 | 0.014 | 0.0259 | 2.43 | 0.0169 | 0.0320 |
| 15 | 11.9 | 10 | 50 | 0.98 | 0.057 | 0.0720 | 0.31 | 0.0533 | 0.0695 |

***S10. Measured* E_cell_ *during experimental runs of Box-Behnken design.***

| Run | Parameters tested | | | E_cell_ (V) |
| --- | --- | --- | --- | --- |
|  | **X_1_^a^** | **X_2_^b^** | **X_3_^c^** |  |
| 1 | 6.6 | 15 | 50 | 35.0 |
| 2 | 1.3 | 15 | 150 | 12.8 |
| 3 | 11.9 | 10 | 250 | 8.5 |
| 4 | 1.3 | 5 | 150 | 6.0 |
| 5 | 6.6 | 5 | 50 | 14.2 |
| 6 | 6.6 | 10 | 150 | 10.7 |
| 7 | 6.6 | 5 | 250 | 5.0 |
| 8 | 11.9 | 5 | 150 | 6.6 |
| 9 | 1.3 | 10 | 250 | 7.0 |
| 10 | 1.3 | 10 | 50 | 19.6 |
| 11 | 6.6 | 10 | 150 | 15.0 |
| 12 | 6.6 | 15 | 250 | 10.2 |
| 13 | 11.9 | 15 | 150 | 17.2 |
| 14 | 6.6 | 10 | 150 | 10.8 |
| 15 | 11.9 | 10 | 50 | 30.0 |
